# Indole-3-Acetic Acid and Skatole Exert Opposing Effects on MDR1 Proteostasis in Human Colonic Epithelial Cells: A Molecular Basis for the Gut Microbial Metabolic Switch

**DOI:** 10.3390/jox16010036

**Published:** 2026-02-18

**Authors:** Kazuma Naito, Ayame Tomii, Katsunori Ishii, Hidehisa Shimizu

**Affiliations:** 1 The United Graduate School of Agricultural Sciences, Tottori University, 4-101 Koyama-Minami, Tottori, Tottori 680-8553, Japan; 2 Graduate School of Natural Science and Technology, Shimane University, 1060 Nishikawatsu-Cho, Matsue, Shimane 690-8504, Japan; 3 Faculty of Life and Environmental Sciences, Shimane University, 1060 Nishikawatsu-Cho, Matsue, Shimane 690-8504, Japan; 4 Estuary Research Center, Shimane University, 1060 Nishikawatsu-Cho, Matsue, Shimane 690-8504, Japan; 5 Interdisciplinary Center for Science Research, Shimane University, 1060 Nishikawatsu-Cho, Matsue, Shimane 690-8504, Japan; 6 Institute of Agricultural and Life Sciences, Academic Assembly, Shimane University, 1060 Nishikawatsu-Cho, Matsue, Shimane 690-8504, Japan

**Keywords:** inflammatory bowel disease, P-glycoprotein, MDR1, aryl hydrocarbon receptor, indole-3-acetic acid, skatole, metabolic switch, proteostasis, xenobiotics, gut microbiota

## Abstract

The escalating consumption of red meat is a potent environmental risk factor for inflammatory bowel disease (IBD), which is characterized by compromised expression of the xenobiotic transporter P-glycoprotein (*MDR1*/*ABCB1*). While gut microbiota metabolize dietary tryptophan into diverse indole derivatives that function as aryl hydrocarbon receptor (AhR) ligands, their differential regulation of MDR1 remains an unresolved AhR paradox. Here, we investigated the mechanisms by which two distinct metabolites, indole-3-acetic acid (IAA) and skatole, regulate MDR1 expression in human colonic epithelial Caco-2 cells. We observed that IAA selectively enhances MDR1 protein stability via an AhR-dependent pathway without inducing de novo transcription, suggesting a mechanism we term enhanced proteostasis mediated by the AhR-Hsp90 complex. Conversely, skatole, a toxic dysbiotic metabolite linked to red meat intake, triggered a time-dependent depletion of MDR1 and potently abrogated the protective efficacy of IAA. Our findings are consistent with a model in which skatole acts as a putative structural disruptor, potentially destabilizing the chaperone complex essential for MDR1 integrity. This destruction is facilitated by a key bacterial enzyme, indoleacetate decarboxylase (IAD), which is a pH-dependent metabolic switch in the gut. The modern Western diet, characterized by high protein and low fiber content, elevates colonic pH, thereby activating IAD to convert protective IAA into toxic skatole. These findings provide a molecular framework for the red meat–microbiome–barrier failure axis and highlight the restoration of the IAA/skatole balance through dietary intervention as a promising therapeutic strategy.

## 1. Introduction

The 21st century has witnessed a dramatic epidemiological shift in inflammatory bowel disease (IBD), including ulcerative colitis (UC) and Crohn’s disease (CD). Once confined largely to Western populations, IBD is now emerging as a global pandemic, with accelerating incidence rates in newly industrialized nations across Asia, South America, and the Middle East [1]. This rapid rise, occurring over a single generation, cannot be explained by genetic drift alone and points unequivocally to environmental drivers. Among these, the Westernization of dietary habits, specifically the pervasive increase in red meat (beef, pork, and lamb) and processed meat consumption, stands out as a primary culprit [2]. A recent landmark dose–response meta-analysis provided compelling evidence that for every 100 g/day increase in red meat intake, the risk of developing UC increases by 65% [3]. Moreover, meat consumption is associated with increased all-cause mortality in patients with IBD [4]. Epidemiological data from Japan reveal a striking generational shift. Meat consumption among adolescents and young adults (aged 15–29 years), particularly males, has surged by approximately 50 g/day over the last two decades [5]. This dietary shift precisely overlaps with the peak age of onset for Crohn’s disease in both the Japanese and US populations [2,6], suggesting that dietary factors act as potent environmental triggers in genetically susceptible populations. Additionally, specific dietary factors reportedly increase the risk of UC relapse [7]. While poultry consumption has also increased and serves as a source of dietary tryptophan [8], epidemiological evidence specifically links red meat to IBD risk [3], suggesting the involvement of additional factors, such as heme iron or saturated fats, in disease pathogenesis.

At the molecular level, the primary event linking dietary overload to chronic inflammation is the collapse of the intestinal epithelial barrier. The epithelium is not merely a physical wall but a dynamic defense system equipped with chemical barriers. Central to this defense is P-glycoprotein (P-gp), encoded by the *MDR1* (*ABCB1*) gene [9]. MDR1 functions as an ATP-dependent efflux pump localized to the apical membrane of enterocytes. Its physiological role extends far beyond drug resistance; it actively extrudes bacterial toxins (e.g., lipid A), pro-inflammatory lipid mediators, and xenobiotics into the intestinal lumen, preventing their accumulation within the mucosa [10]. Clinical evidence shows that MDR1 expression is consistently downregulated in the inflamed mucosa of patients with IBD [11]. In animal models, *Mdr1a*-deficiency leads to spontaneous colitis driven by commensal bacteria, highlighting that MDR1 dysfunction is a primary cause, rather than just a consequence, of inflammation [12,13]. Recent insights also suggest a bidirectional relationship in which drug transporters, such as MDR1, regulate the gut microbiota and inflammation [14], and dysbiosis is intricately linked to colorectal cancer pathogenesis [15]. Therefore, identifying the dietary and microbial factors that regulate MDR1 stability is critical for preventing IBD progression.

Expanding on the dietary links, the gut microbiota serves as a metabolic interface between diet and the host, playing a crucial role in mediating these effects. Metabolomic analyses of patients with IBD and animal models have revealed distinct alterations in tryptophan metabolism. Specifically, these studies reported a significant reduction in protective indole derivatives, such as IAA, concurrent with the accumulation of detrimental metabolites, such as skatole [16,17], directly linking these metabolic shifts to disease pathogenesis. Tryptophan, an essential amino acid abundant in animal proteins, is a key precursor of microbial metabolism. Commensal bacteria convert unabsorbed tryptophan into a myriad of indole derivatives [18], which serve as endogenous ligands for the host aryl hydrocarbon receptor (AhR) [19]. Evolutionarily, AhR did not evolve to sense industrial toxins such as dioxin (TCDD), but rather to detect dietary and microbial metabolites to maintain barrier homeostasis and immune tolerance. It acts as a sensor of the chemical environment of the gut [20,21]. However, modern meat-heavy diets have distorted this ancient communication channel, leading to an evolutionary mismatch. While our ancestors relied on plant-based signals (e.g., IAA from fiber fermentation), the modern gut is flooded with meat-derived signals (e.g., skatole).

A confounding aspect of AhR biology is the AhR paradox: different ligands binding to the same receptor can elicit diametrically opposing biological outcomes. While some ligands promote regulatory T cell (Treg) differentiation and barrier repair, others induce pro-inflammatory Th17 responses or cellular toxicity [19,22,23]. In this study, we aimed to elucidate this paradox by focusing on two contrasting tryptophan metabolites that dominate the gut environment under different dietary conditions:

- IAA: Produced by beneficial commensals such as *Bifidobacterium* and *Bacteroides*, IAA is generally regarded as a protective metabolite that maintains epithelial integrity through IL-22 production [23]. Furthermore, recent research has highlighted its role in reprogramming systemic homeostasis to ameliorate IBD-associated systemic complications such as cachexia, independent of food intake [24]. IAA has also been reported to inhibit cell proliferation and TNF-α expression via pathways such as that of TLR4 [25,26].
- Skatole (3-methylindole): A malodorous metabolite produced from IAA by specific dysbiotic bacteria (e.g., *Clostridium scatologenes*) via decarboxylation [27,28]. This conversion is mediated by indoleacetate decarboxylase (IAD), which is sensitive to the gut chemical environment. Skatole production is favored under conditions of high protein availability and elevated colonic pH [29]. It is associated with cytotoxicity and foul odor [30,31], and its metabolism involves specific cytochrome P450 enzymes [31,32]. Unlike protective indoles, our research group has demonstrated that skatole directly triggers inflammatory responses in intestinal epithelial cells. Specifically, skatole upregulates the expression of pro-inflammatory cytokines, such as IL-6 and TNF-α, via the activation of p38 MAPK and NF-κB signaling pathways [33,34,35], thereby actively contributing to mucosal inflammation and IBD pathology.

Despite sharing the indole scaffold, why does IAA protect the gut, whereas skatole damages it? However, the specific impact of these metabolites on MDR1 quality control remains unclear. To resolve this paradox, we introduced the concept of selective AhR modulator (SAhRM) properties [36]. We hypothesize that the subtle structural differences between IAA (possessing an acidic side chain, -CH_2_COOH) and skatole (a planar, hydrophobic molecule with a methyl group, -CH_3_) dictate the distinct conformational changes in the AhR protein upon ligand binding. This conformational shape determines not only the transcriptional activity but, more importantly, the interaction of the receptor with cytosolic chaperone networks. Crucially, in the cytoplasm, unliganded AhR is stabilized by a chaperone complex containing Heat Shock Protein 90 (Hsp90), XAP2, and p23 [37]. MDR1 is also an obligate Hsp90 client protein that requires this chaperone for proper folding, membrane trafficking, and protection from ubiquitin-mediated degradation [38]. Conversely, potent AhR activation by toxic ligands (such as TCDD) can trigger a non-canonical pathway in which AhR functions as a component of the CUL4B E3 ubiquitin ligase complex, actively targeting proteins for proteasomal degradation [39].

Based on these observations and drawing upon established AhR–chaperone biology, we propose and explore a tug-of-war model for MDR1 stability:

1. IAA acts as a safe SAhRM that stabilizes the AhR-Hsp90 complex, thereby increasing the pool of chaperones available to protect MDR1 (enhanced proteostasis).
2. We propose that skatole acts as a structural disruptor that competitively displaces IAA and destabilizes the chaperone complex, actively accelerating MDR1 degradation. Furthermore, we explored the clinical significance of the bacterial enzyme indoleacetate decarboxylase (IAD). We propose that IAD acts as a metabolic switch that converts protective IAA into toxic skatole [27,29], thereby flipping AhR function from protection to destruction.

The present study aims to provide a unified molecular theory linking the red meat–microbiome–barrier failure axis, offering a mechanistic basis for novel therapeutic interventions.

## 2. Materials and Methods

### 2.1. Materials

The reagents and antibodies used in this study were obtained from the following sources: anti-β-actin antibody (C4) was purchased from Santa Cruz Biotechnology Inc. (Dallas, TX, USA). Anti-P-glycoprotein (MDR1) polyclonal antibody was purchased from Proteintech Group Inc. (Chicago, IL, USA). Secondary antibodies, specifically Peroxidase AffiniPure Goat Anti-Rabbit IgG (H + L) and Anti-Mouse IgG (H + L), were supplied by Jackson ImmunoResearch Laboratories, Inc. (West Grove, PA, USA). Indole-3-acetic acid (IAA; ≥98.0%), protease inhibitor cocktail (EDTA-free), and phosphatase inhibitor cocktail were sourced from Nacalai Tesque Inc. (Kyoto, Japan). 3-Methylindole (skatole; >98.0%) was obtained from Tokyo Chemical Industry Co., Ltd. (Tokyo, Japan). Cell culture reagents, including penicillin–streptomycin solution and high-glucose Dulbecco’s modified Eagle’s medium (DMEM), were obtained from Wako Pure Chemical Industries, Ltd. (Osaka, Japan). The AhR antagonist CH-223191 was purchased from Cayman Chemical (Ann Arbor, MI, USA), and fetal bovine serum (FBS) was obtained from Biowest S.A.S. (Nuaillé, France).

### 2.2. Cell Culture

Human colorectal adenocarcinoma Caco-2 cells (provided by RIKEN Cell Bank, Tsukuba, Japan) were cultured in high-glucose DMEM containing 10% FBS, 100 U/mL penicillin, and 100 µg/mL streptomycin. The cultures were maintained at 37 °C in a humidified incubator with 5% CO_2_. Prior to the assays, the cells were switched to serum-free DMEM for a 24 h starvation period. Dimethyl sulfoxide (DMSO) was used as the solvent for IAA, skatole, and CH-223191, with the final DMSO concentration in the culture medium maintained at 0.1% (*v*/*v*) for all treatment groups, including the vehicle control. We selected a skatole concentration of 1000 µM to replicate the pathological fecal levels observed under dysbiotic conditions (~1080 µM) [31]. Previous validation confirmed that this concentration does not trigger significant cytotoxicity (Caspase-3/7 activation) within the exposure times used [33]. An equimolar concentration of IAA (1000 µM) was used to enable direct comparison and mimic physiological intracolonic levels [31].

### 2.3. Quantitative Real-Time PCR

For transcriptional analysis, serum-starved Caco-2 cells were pre-treated with either vehicle (0.1% DMSO) or the AhR antagonist CH-223191 (10 µM) for 30 min. This was followed by stimulation with vehicle, IAA (1000 µM), or skatole (1000 µM) for periods of 1, 3, or 6 h. RNA extraction was performed using the phenol–chloroform-based Sepasol-RNA I Super G system (Nacalai Tesque Inc., Kyoto, Japan) according to the manufacturer’s protocol [40]. cDNA synthesis was carried out using 1 µg of total RNA template and PrimeScript™ RT Master Mix (Perfect Real Time) (Takara Bio Co. Inc., Shiga, Japan). Gene expression was quantified via real-time PCR using TB Green™ Premix Ex Taq™ II (Tli RNaseH Plus) on a Thermal Cycler Dice Real-Time System III (Takara Bio Co. Inc., Shiga, Japan). The specific primer sequences are presented in Table 1. Relative mRNA abundance was calculated using the calibration curve method, with values normalized to that of the reference gene, *RPLP0*.

### 2.4. Immunoblotting

Following serum starvation, cells were pre-incubated with vehicle or 10 µM CH-223191 for 30 min and subsequently exposed to IAA (1000 µM), skatole (1000 µM), or vehicle for the designated times. Whole-cell protein lysates were generated using 1% NP-40 lysis buffer (150 mM NaCl, 50 mM Tris-HCl, 10% glycerol) supplemented with protease and phosphatase inhibitor cocktails. Lysates were clarified by centrifugation (15,000× *g* for 10 min at 4 °C) as previously described [41]. Protein samples were resolved by SDS-PAGE and transferred to Immobilon-P PVDF membranes (Millipore Inc., Bedford, MA, USA). Membranes were probed with anti-MDR1 antibody (1:5000 dilution) and anti-β-actin antibody (1:5000 dilution). Secondary detection was performed using HRP-conjugated Goat Anti-Rabbit or Anti-Mouse IgG (1:5000 dilution). Signals were visualized using the Chemi-Lumi One L system (Nacalai Tesque Inc., Kyoto, Japan) and captured with an ImageQuant™ LAS 4010 (GE Healthcare Life Sciences, Marlborough, MA, USA). Densitometric analysis was conducted using ImageJ software (https://imagej.net/ij/; NIH, Bethesda, MD, USA) with a Band/Peak Quantification macro [42], with MDR1 signals normalized to β-actin (Representative uncropped Western blot images are shown in Supplementary Figure S1.).

### 2.5. Statistical Analysis

**Figure 1:**
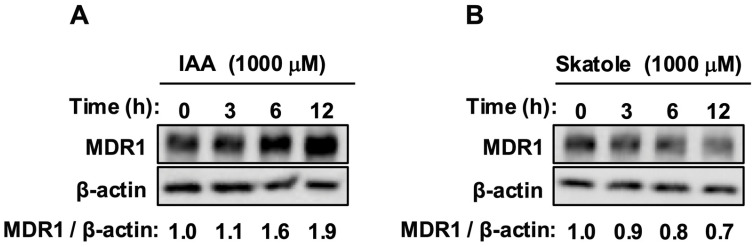
IAA increased MDR1 protein levels, whereas skatole reduced them: (**A**) Caco-2 cells were treated with IAA (1000 µM) for 3, 6, and 12 h. IAA treatment induced a time-dependent increase in MDR1 protein. (**B**) Cells were treated with skatole (1000 µM) for 3, 6, and 12 h. Skatole treatment induced a time-dependent decrease in the expression of MDR1 protein. Protein levels were analyzed using Western blotting. The numerical values below the bands represent the mean intensity of MDR1 normalized to β-actin from three independent experiments. 0 h, time zero control; IAA, indole-3-acetic acid.

**Figure 2:**
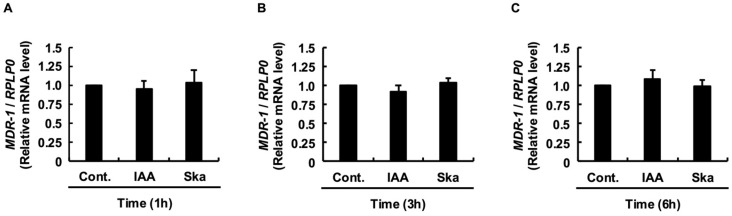
Neither IAA nor skatole induced *MDR1* mRNA expression. Relative mRNA expression of *MDR1* in cells treated with IAA or skatole for 1 h (**A**), 3 h (**B**), and 6 h (**C**). Data are presented as the mean ± SE of four independent experiments. Statistical significance was evaluated using one-way ANOVA, followed by the Tukey–Kramer test (not significant vs. control). Cont., control (DMSO); IAA, indole-3-acetic acid.

**Figure 3:**
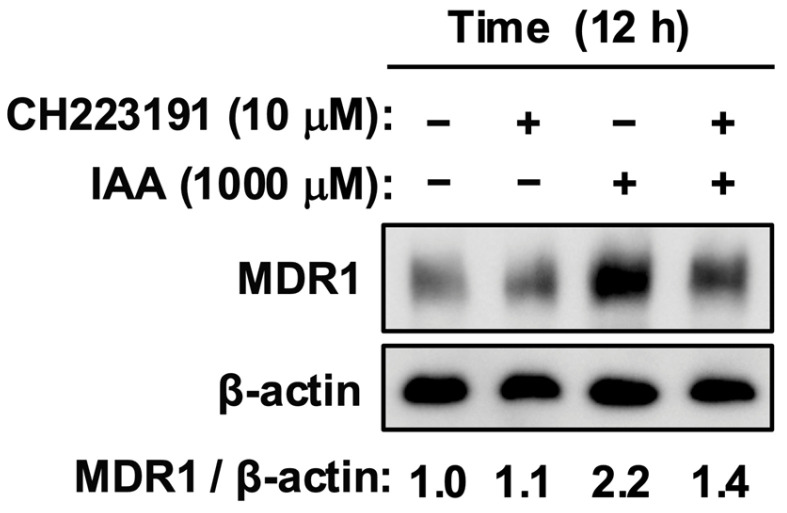
AhR antagonism attenuates IAA-mediated MDR1 stabilization. Cells were pretreated with the AhR antagonist CH-223191 (10 µM), followed by treatment with IAA. The antagonist suppressed the IAA-induced increase in MDR1 expression. Protein levels were analyzed by Western blotting. The numerical values below the bands represent the mean intensity of MDR1 normalized to β-actin from three independent experiments. −, vehicle control; IAA, indole-3-acetic acid.

**Figure 4:**
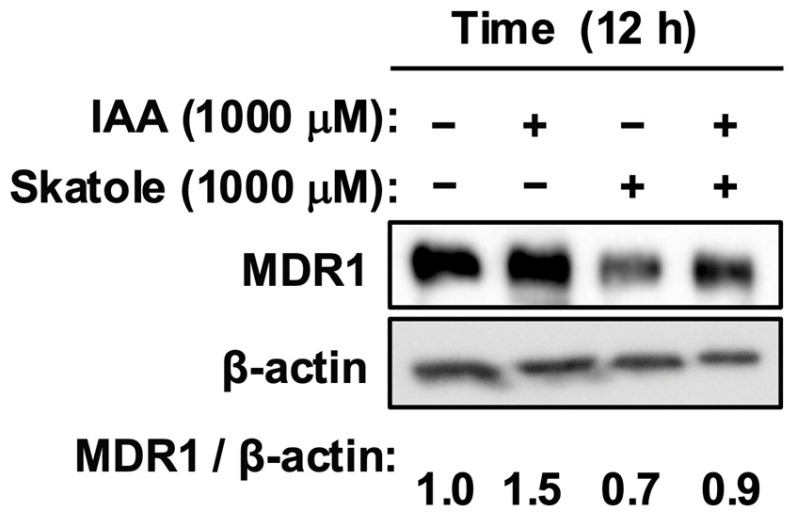
Skatole inhibited the protective effect of IAA and reduced MDR1 levels. Cells were treated with IAA alone or co-treated with skatole (1000 µM). Skatole treatment completely nullified the IAA-induced increase, reducing MDR1 protein levels compared to the IAA group. Protein levels were analyzed by Western blotting. Band intensity of MDR1 was normalized to β-actin. The numerical values below the bands represent the mean intensity of MDR1 normalized to β-actin from three independent experiments. −, vehicle control; IAA, indole-3-acetic acid.

Representative results for Western blot analyses are presented in Figure 1 as the mean values of three independent biological replicates. For the quantitative real-time PCR data (Figure 2), results are expressed as the mean ± standard error (SE) derived from four independent biological replicates. Statistical significance for Figure 2 was evaluated using one-way ANOVA followed by the Tukey–Kramer post hoc test. Additional Western blot results are presented in Figure 3 and Figure 4, showing the mean values of three independent biological replicates. All analyses were performed using Microsoft Excel 2021 (Microsoft Corp., Redmond, WA, USA) and Statcel 4 (OMS Publishing Co., Saitama, Japan). Statistical significance was set at *p* < 0.05.

## 3. Results

### 3.1. IAA Enhances MDR1 Protein Stability, Whereas Skatole Triggers Its Depletion

We first evaluated the effects of IAA and skatole on MDR1 protein expression. IAA treatment (1000 µM) induced a substantial, time-dependent accumulation of MDR1 protein, peaking at 12 h (Figure 1A). In contrast, treatment with skatole at equimolar concentration resulted in a progressive reduction in MDR1 protein levels relative to the controls (Figure 1B). These data indicate that while IAA functions as a positive regulator of MDR1 stability, skatole has a destabilizing effect.

### 3.2. Uncoupling of Protein and mRNA: Evidence for Post-Translational Regulation

To ascertain whether these changes were transcriptionally driven, we quantified the MDR1 mRNA levels. Despite the profound alterations in protein abundance, quantitative PCR analysis revealed that neither IAA nor skatole significantly altered *MDR1* mRNA expression at 1, 3, or 6 h compared to controls (*p* > 0.05; Figure 2A–C). This complete uncoupling of protein levels from gene expression strongly supports a post-translational mechanism—specifically, IAA-mediated stabilization versus skatole-induced turnover.

### 3.3. IAA-Mediated Stabilization Is Strictly AhR-Dependent

The concentration of CH-223191 (10 µM) was selected based on our previous study, which demonstrated its potency to effectively inhibit AhR-mediated signaling in Caco-2 cells [25,32]. Next, we investigated the requirement for AhR signaling using the selective antagonist CH-223191. Pretreatment with CH-223191 (10 µM) markedly attenuated IAA-induced accumulation of MDR1 protein (Figure 3). This finding confirms that AhR engagement is indispensable for the protein-stabilizing efficacy of IAA.

### 3.4. Skatole Acts as a Functional Antagonist to Abrogate the Protective Effect of IAA

To mimic a dysbiotic gut environment in which both metabolites co-occur, we performed co-treatment assays. While IAA alone potentiated MDR1 levels, the concurrent addition of skatole (1000 µM) strongly suppressed this increase, reducing MDR1 expression to levels below those observed in the IAA-treated group (Figure 4). This demonstrates that skatole acts as a potent functional antagonist, effectively overriding the protective signal conferred by IAA.

## 4. Discussion

**Figure 5:**
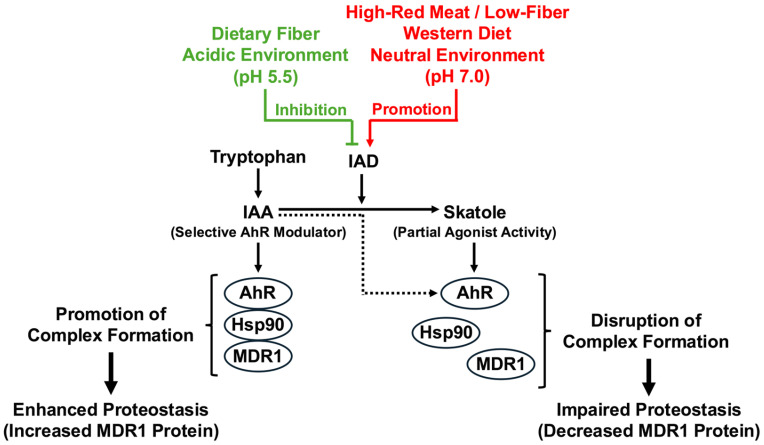
Schematic representation of the proposed metabolic switch and MDR1 proteostasis. (Left) healthy state: Consumption of dietary fiber maintains an acidic colonic environment (pH 5.5), inhibiting the bacterial enzyme indoleacetate decarboxylase (IAD). Consequently, tryptophan is metabolized to indole-3-acetic acid (IAA). IAA acts as a selective AhR modulator that stabilizes the AhR-Hsp90 complex, thereby promoting the folding and stability of MDR1 (enhanced proteostasis). (Right) dysbiotic state (Western diet): High intake of red meat and low fiber creates a neutral environment (pH 7.0), activating IAD. This metabolic switch converts IAA into skatole. Skatole acts as a partial agonist and structural disruptor, destabilizing the chaperone complex and leading to MDR1 degradation (impaired proteostasis). AhR, aryl hydrocarbon receptor; Hsp90, heat shock protein 90; IAA, indole-3-acetic acid.

In the present study, using a human colonic epithelial cell model, we demonstrated that the gut bacterial tryptophan metabolites IAA and skatole exert opposing effects on the critical barrier factor MDR1. Key findings are as follows: (1) IAA enhances MDR1 protein stability via an AhR-dependent pathway without transcriptional induction (enhanced proteostasis); (2) skatole reduces MDR1 protein levels and negates the protective effect of IAA; and (3) neither metabolite alters *MDR1* mRNA expression. A schematic summary of these key findings is shown in Figure 5. These results suggest that metabolic switching in the gut environment is deeply involved in the pathogenesis of IBD.

### 4.1. The SAhRM Concept: Structure Dictates Destiny in AhR Signaling

Our findings challenge the binary view of AhR ligands (agonist vs. antagonist) and provide a mechanistic resolution to the AhR paradox through the lens of the selective AhR modulator (SAhRM) concept [36]. We propose that the structural code of the ligand dictates the functional output of the receptor. The structural distinction is subtle but decisive: IAA possesses a flexible, hydrophilic, acidic side chain (-CH_2_COOH), whereas skatole is a rigid, planar, and hydrophobic molecule (-CH_3_). Based on structural modeling studies [43], we hypothesized that this difference induces distinct conformational changes in the AhR ligand-binding pocket (LBP). We hypothesized that the flexible nature of IAA may induce moderate conformational changes. Although direct biophysical interaction studies (e.g., co-immunoprecipitation) were not within the scope of this study, our data are strongly supported by the established canonical behavior of AhR. The unliganded AhR is stabilized by a chaperone complex containing Hsp90 [37]. Crucially, our observation that IAA increases MDR1 protein levels without affecting mRNA levels (Figure 2) is mechanistically consistent with the stabilization of this Hsp90–client complex. Unlike high-affinity toxins (e.g., TCDD), which trigger rapid AhR nuclear translocation and can even convert AhR into a CUL4B E3 ubiquitin ligase component, promoting protein degradation [39], we posit that IAA preserves the chaperone pool. This mechanism, which we term enhanced proteostasis, explains why MDR1 protein levels increase without a corresponding increase in mRNA levels. In contrast, previous studies have characterized skatole as a partial aryl hydrocarbon receptor agonist [44,45,46]. We propose that this partial agonism reflects an intermediate conformational state of AhR that is sufficient to induce limited transcription (e.g., *CYP1A1*) but structurally incompetent to maintain the stable chaperone complex required for MDR1 proteostasis. Thus, while classical pharmacology focuses on transcriptional potency, our data highlight that for MDR1 regulation, the quality of the ligand–receptor–chaperone interaction is more critical than the quantity of transcriptional induction.

### 4.2. IAA: The Safe Agonist and Enhanced Proteostasis

Our most striking finding was that IAA robustly accumulated MDR1 protein without inducing de novo transcription at any time point tested (Figure 2). This uncoupling of protein levels from mRNA levels suggests a mechanism of enhanced proteostasis in the cells. MDR1 is a large (170 kDa) transmembrane protein that is notoriously difficult to fold and to maintain. It is an obligate client protein of the chaperone Hsp90 [38]. Crucially, unliganded AhR also resides in a cytosolic complex with Hsp90 [37]. We propose that IAA acts as a safe, weak agonist. Unlike high-affinity toxins such as TCDD, which trigger rapid AhR nuclear translocation and can even convert AhR into a CUL4B E3 ubiquitin ligase component, promoting protein degradation [39], IAA likely avoids this toxic switch. Instead, it appears to stabilize the AhR-Hsp90 complex, thereby maintaining a local reservoir of chaperones available to protect MDR1 from ubiquitin-mediated degradation. Although we did not directly measure transport activity, previous studies have established a linear correlation between MDR1 protein abundance and functional efflux capacity (e.g., Rhodamine 123 transport) in Caco-2 cells [10,12]. Since MDR1 acts as an apical efflux pump, its functional capacity is fundamentally rate-limited by the density of transporter units on the membrane [9]. Thus, IAA-mediated stabilization of the MDR1 protein pool directly enhances the detoxification potential of the epithelial barrier. Furthermore, our previous findings demonstrated that IAA exerts anti-inflammatory effects via TLR4 inhibition [25,26]. Since inflammation (e.g., TNF-α) itself can precipitate barrier dysfunction, IAA likely preserves MDR1 through a dual mechanism: direct stabilization via the AhR-Hsp90 axis and indirect protection by dampening TLR4-mediated inflammatory insults. Additionally, a recent study demonstrated that IAA promotes mucin sulfation to maintain intestinal homeostasis [47]. Together with our findings on MDR1, this suggests that IAA functions as a master regulator of the epithelial barrier, orchestrating a multi-layered defense system involving both xenobiotic efflux (MDR1) and physical shielding (mucins).

### 4.3. Skatole: A Putative Structural Disruptor

In stark contrast to IAA, skatole treatment reduced MDR1 protein levels (Figure 1B) and completely abolished the protective effect of IAA (Figure 4). A critical question is whether this reduction merely reflects nonspecific cytotoxicity. However, our immunoblotting data were normalized to β-actin, a fundamental cytoskeletal protein. The selective reduction in the MDR1/β-actin ratio indicates that MDR1 is preferentially degraded over general cellular proteins. If nonspecific necrosis were the primary driver, β-actin levels would likely decline in parallel, maintaining a constant ratio. Furthermore, we previously demonstrated that skatole at this concentration does not significantly increase Caspase-3/7 activity in Caco-2 cells within 12 h [33]. This rules out extensive cell death as the primary cause of the rapid MDR1 downregulation observed in the present study. The toxicity of skatole appears to be driven by the combined effects of structural antagonism and inflammation. First, regarding structural antagonism, the AhR ligand-binding pocket is highly hydrophobic in nature. Skatole, which is more hydrophobic than IAA, likely binds with a higher affinity, potentially displacing protective IAA [43,45]. Although further structural studies are required to visualize the physical displacement of Hsp90, our functional data demonstrating that AhR antagonism (Figure 3) and skatole co-treatment (Figure 4) modulate MDR1 protein levels mirror the expected behavior of a ligand-dependent chaperone interaction. We infer that its rigid planar structure may be insufficient to retain Hsp90, or it may physically displace Hsp90 from the ligand-binding domain, as suggested in other AhR ligand studies [48]. Deprived of Hsp90 support, MDR1 is likely exposed to ubiquitination by specific E3 ligases (e.g., FBXO15 or NEDD4-1) and is rapidly degraded [38,49]. It is important to note that we avoided the use of proteasome inhibitors (e.g., MG132) in this study because AhR itself is rapidly degraded by the proteasome upon activation [39]. Global proteasome inhibition results in the artificial accumulation of activated AhR, confounding the interpretation of the specific chaperone-sharing mechanism. Therefore, our data provide a clear demonstration of ligand-dependent proteostasis modulation. Furthermore, the differential effects of IAA and skatole on MDR1 stability may be partially attributed to their distinct electrochemical properties. Electrochemical analyses have revealed that while IAA exhibits a relatively low oxidation potential (~+0.6 V at pH 7.0) and undergoes oxidative decarboxylation [50], skatole shows a higher oxidation potential (~+0.8 V). Crucially, due to the presence of the C3-methyl group, skatole oxidation does not allow for decarboxylation but instead proceeds via oxidative dehydrogenation to generate 3-methyleneindolenine (3MEIN) [32]. 3MEIN is a potent electrophile known to form covalent adducts with cellular protein nucleophiles (e.g., cysteine residues). We propose that this skatole-derived electrophile may chemically modify and destabilize the AhR-Hsp90-MDR1 chaperone complex, thereby accelerating ubiquitin-mediated degradation, a toxicity pathway not triggered by IAA. Second, regarding inflammatory signals, we previously reported that skatole activates the p38 MAPK and NF-κB pathways, inducing the expression of inflammatory cytokines (IL-6 and TNF-α) [34,35]. These inflammatory signals further compromise epithelial homeostasis and accelerate barrier dysfunction [51].

### 4.4. Physiological Relevance of Skatole Concentrations

A potential concern in in vitro studies is the physiological relevance of the ligand concentrations. In the present study, we used 1000 µM skatole. While this concentration may seem high compared to healthy conditions, it is highly relevant in pathological states. Intestinal skatole production is strictly dependent on dietary tryptophan availability [20]. Importantly, a recent review calculated that while fecal skatole concentrations in healthy individuals are low (~54 µM), they can reach approximately 1080 µM in patients with disturbed digestion or dysbiosis [31]. This directly validates our use of 1000 µM as a clinically relevant concentration that mimics the dysbiotic gut environment. IAA is a direct precursor of skatole and is generally present in the intestine in much higher amounts than skatole [32]. Therefore, under conditions in which the IAD enzyme is inactivated (e.g., low pH), conversion to skatole is blocked, leading to the preservation and accumulation of IAA at millimolar levels, which supports the physiological relevance of the IAA concentration used in the present study.

### 4.5. Clinical Implications: The Metabolic Switch and Double Hit

Our study highlights the bacterial enzyme indoleacetate decarboxylase (IAD) as a pivotal metabolic switch that regulates host barrier integrity. The progression from a healthy gut to IBD may be viewed as a malfunction of this switch, driven by a modern Westernized diet. High-fat, high-protein diets reportedly induce dysbiosis and alter tryptophan metabolism [8,52]. We propose that this creates a perfect storm for toxic skatole production via a double-hit mechanism regulating IAD:

1. Loss of the acidic brake (enzymatic regulation): IAD is a pH-sensitive enzyme; its activity is suppressed under acidic conditions but hyperactivated in neutral/alkaline environments [29,31]. In a healthy gut, fermentation of dietary fiber yields short-chain fatty acids (SCFAs), maintaining an acidic pH that inhibits IAD. However, red meat consumption produces ammonia, elevating colonic pH and releasing this brake.
2. Activation by carbon catabolite repression (genetic regulation): When fermentable fibers are scarce (low-fiber diet), bacteria are forced to switch their metabolism from sugars to amino acids, genetically inducing IAD expression to utilize tryptophan as an energy source [27]. Biochemically, IAD belongs to the glycyl radical enzyme (GRE) family [27], which requires strict anaerobic conditions for activation. This suggests that skatole production is confined to specific niches within the dysbiotic gut, where oxygen is depleted, and pH is elevated.

The ‘metabolic switch’ mediated by the bacterial enzyme IAD is pH-dependent. It is crucial to distinguish luminal events from intracellular signaling. While luminal pH fluctuates, intestinal epithelial cells strictly maintain intracellular pH (pHi) at 7.2–7.4 via transporters such as NHE3 [53,54]. Thus, our in vitro conditions at physiological pH accurately mimic the intracellular environment where AhR signaling occurs. Clinically, although both red meat and poultry (white meat) provide tryptophan substrates for skatole production [8], epidemiological evidence strongly links red meat to IBD risk [3]. We postulated a ‘double-hit’ model: red meat provides both the substrate (tryptophan) for skatole and the inflammatory cofactors (heme iron, saturated fats) that exacerbate barrier damage, whereas poultry lacks these aggravating factors. Importantly, this suggests that therapeutic strategies need not act directly on the host immune system but can also target the luminal environment. By restoring the acidic brake, for example, by consuming foods rich in fermentable fibers, specifically resistant starch [55], to lower colonic pH, it may be possible to effectively inhibit skatole formation. Indeed, nutritional intervention studies have confirmed that the inclusion of fermentable fiber or resistant starch significantly suppresses skatole production in the colon [55,56] and the development of colitis [57]. This offers a scientifically grounded molecular rationale for dietary interventions as first-line therapy for maintaining remission in IBD.

### 4.6. Limitations

The present study had several limitations that warrant discussion. First, although the Caco-2 cell line provides a valuable and reproducible model for studying intestinal epithelial function, it is a transformed cancer cell line that represents a simplified monoculture system. Consequently, it may not fully recapitulate the complex physiological environment of the native intestinal epithelium. Specifically, the absence of other critical cell types, such as immune cells, goblet cells, or Paneth cells, as well as the intricate tissue architecture and interactions with the gut microbiota, limits the direct translation of these findings to the in vivo setting. Future studies incorporating co-culture systems or organoid models, in addition to in vivo verification, will provide a more holistic understanding of MDR1 regulation. Second, beyond the physical interaction of the AhR-Hsp90-MDR1 complex, our proposed selective AhR modulator (SAhRM) concept is based on the idea that IAA and skatole induce distinct conformational changes in the AhR ligand-binding pocket, dictating their functional outcomes. While indirect functional evidence and established AhR biology support this, direct structural or biophysical studies, such as X-ray crystallography, NMR, or molecular dynamics simulations, are required to visualize these precise conformational changes and the proposed competitive displacement of IAA by skatole, thereby definitively validating the structural disruptor model. Third, our study proposes a pivotal role for the bacterial enzyme indoleacetate decarboxylase (IAD) as a metabolic switch in the gut microbiome. However, our in vitro Caco-2 model inherently lacks the complex microbial community and dynamic enzymatic activity of the gut microbiome. While we inferred the activity of IAD based on dietary conditions and pH logic, direct experimental validation of IAD activity modulation by dietary factors (e.g., pH and substrate availability) within a microbial community was beyond the scope of this cell-based study. Future research should aim to integrate microbial components to directly assess the proposed mechanisms in a more physiologically relevant context. Fourth, although the concentrations of skatole (1000 µM) and IAA (1000 µM) used in this study are justified by their reported levels in pathological or certain physiological conditions, respectively [32], the gut lumen exhibits a wide and fluctuating range of metabolites. Our study utilized fixed, relatively high concentrations to investigate the distinct mechanisms. Furthermore, the 12 h duration of our experiments focused on the acute molecular initiation of MDR1 destabilization. Although IBD is a chronic disease, identifying the immediate triggers of barrier dysfunction is crucial for understanding disease onset. Future long-term exposure studies or chronic animal models would be beneficial for fully elucidating the long-term consequences of these metabolic interactions. Finally, the gut microbiota produces a diverse array of tryptophan-derived indole metabolites that act as AhR ligands (e.g., indole, indole-3-propionic acid, and indole-3-aldehyde), each potentially exhibiting unique SAhRM properties. Our study focused specifically on IAA and skatole because of their contrasting effects and relevance to IBD pathology. The complex interplay between these and other microbial indoles has not been investigated and remains an important avenue for future research to understand the overall AhR signaling landscape in the gut.

## 5. Conclusions

The present study resolves the AhR paradox in gut immunity by demonstrating that the ligand structure dictates the fate of the intestinal barrier. Our study suggests that IAA functions as a stabilizer of MDR1 proteostasis via the AhR-Hsp90 axis, whereas skatole acts as a structural disruptor. The bacterial IAD enzyme, which converts protective IAA into toxic skatole under dysbiotic conditions, represents a pivotal checkpoint linking the Western diet to IBD. Therefore, IBD management must evolve beyond downstream immune suppression to upstream metabolic normalization, targeting the gut environment to restore the dominance of protective IAA.

## Figures and Tables

**Table 1:** Forward (Fw) and reverse (Rv) primers were used for the target genes.

Target Genes	GenBank Accession No.	Primers (5 → 3′)	Location	Length (bp)	Product Length (bp)	Primer Conc. (nM)
*MDR1*	NM_001348945.2	Fw: TTGCTGCTTACATTCAGGTTTCA	897–919	23	105	250
		Rv: AGCCTATCTCCTGTCGCATTA	1001–981	21		250
*RPLP0*	NM_001002.4	Fw: CGACCTGGAAGTCCAACTAC	97–116	20	108	250
		Rv: ATCTGCTGCATCTGCTTG	205–188	18		250

## Data Availability

The original contributions presented in this study are included in this article/Supplementary Material. Further inquiries can be directed to the corresponding author.

## Supplementary Materials

The following supporting information can be downloaded at https://www.mdpi.com/article/10.3390/jox16010036/s1, Supplementary file: Uncropped Western blot images.

## References

[1] Ng S.C., Shi H.Y., Hamidi N., Underwood F.E., Tang W., Benchimol E.I., Panaccione R., Ghosh S., Wu J.C.Y., Chan F.K.L. (2017). Worldwide incidence and prevalence of inflammatory bowel disease in the 21st century: A systematic review of population-based studies. Lancet.

[2] Matsuoka K., Fujii T., Okamoto R., Yamada A., Kunisaki R., Matsuura M., Watanabe K., Shiga H., Takatsu N., Bamba S. (2022). Characteristics of adult patients newly diagnosed with Crohn’s disease: Interim analysis of the nation-wide inception cohort registry study of patients with Crohn’s disease in Japan (iCREST-CD). J. Gastroenterol..

[3] Zhang Y., Yu Y., Jiang Z., Yu J., Zhang Z., An Z., Du Y., Mao Y., Hu L., Tang X. (2025). The impact of red meat and processed meat consumption on the risk of development and relapse of ulcerative colitis: A systematic review and dose-response meta-analysis. Front. Nutr..

[4] Chen H., Fu T., Dan L., Chen X., Sun Y., Chen J., Wang X., Hesketh T. (2022). Meat consumption and all-cause mortality in 5763 patients with inflammatory bowel disease: A retrospective cohort study. eClinicalMedicine.

[5] Shinsugi C., Takimoto H. (2025). Food Consumption Trends in Japanese Children and Adolescents: The National Health and Nutrition Survey, 2001–2019. Foods.

[6] Nakajo K., Yamazaki M., Chung H., Xu Y., Qiu H. (2024). Trends in the prevalence and incidence of Crohn’s disease in Japan and the United States. Int. J. Color. Dis..

[7] Jowett S.L., Seal C.J., Pearce M.S., Phillips E., Gregory W., Barton J.R., Welfare M.R. (2004). Influence of dietary factors on the clinical course of ulcerative colitis: A prospective cohort study. Gut.

[8] Shi J., Zhao D., Zhao F., Wang C., Zamaratskaia G., Li C. (2021). Chicken-eaters and pork-eaters have different gut microbiota and tryptophan metabolites. Sci. Rep..

[9] Thiebaut F., Tsuruo T., Hamada H., Gottesman M.M., Pastan I., Willingham M.C. (1987). Cellular localization of the multi-drug-resistance gene product P-glycoprotein in normal human tissues. Proc. Natl. Acad. Sci. USA.

[10] Ho G.T., Moodie F.M., Satsangi J. (2003). Multidrug resistance 1 gene (P-glycoprotein 170): An important determinant in gastrointestinal disease?. Gut.

[11] Ho G.T., Plevy S.E., Satsangi J. (2003). MDR1 gene mutation, mRNA expression and P-glycoprotein expression in inflammatory bowel disease. Gut.

[12] Panwala C.M., Jones J.C., Viney J.L. (1998). A novel model of inflammatory bowel disease: Mice deficient for the multiple drug resistance (Mdr1a) gene develop spontaneous colitis. J. Immunol..

[13] Cao W., Kayama H., Chen M.L., Delmas A., Sun A., Kim S.Y., Rangarajan E.S., McKevitt K., Beck A.P., Jackson C.B. (2017). The Xenobiotic Transporter Mdr1 Enforces T Cell Homeostasis in the Presence of Intestinal Bile Acids. Immunity.

[14] Stoeltje L., Luc J.K., Haddad T., Schrankel C.S. (2024). The roles of ABCB1/P-glycoprotein drug transporters in regulating gut microbes and inflammation: Insights from animal models, old and new. Philos. Trans. R. Soc. Lond. B Biol. Sci..

[15] Brennan C.A., Garrett W.S. (2016). Gut Microbiota, Inflammation, and Colorectal Cancer. Annu. Rev. Microbiol..

[16] Shiomi Y., Nishiumi S., Ooi M., Hatano N., Shinohara M., Yoshie T., Kondo Y., Furumatsu K., Shiomi H., Kutsumi H. (2011). GCMS-based Metabolomic Study in Mice with Colitis Induced by Dextran Sulfate Sodium. Inflamm. Bowel Dis..

[17] Lai Y., Xue J., Liu C.W., Gao B., Chi L., Tu P., Lu K., Ru H. (2019). Serum Metabolomics Identifies Altered Bioenergetics, Signaling Cascades in Parallel with Exposome Markers in Crohn’s Disease. Molecules.

[18] Roager H.M., Licht T.R. (2018). Microbial tryptophan catabolites in health and disease. Nat. Commun..

[19] Stockinger B., Di Meglio P., Gialitakis M., Duarte J.H. (2014). The aryl hydrocarbon receptor: Multitasking in the immune system. Annu. Rev. Immunol..

[20] Yokoyama M.T., Carlson J.R. (1979). Microbial metabolites of tryptophan in the intestinal tract with special reference to skatole. Am. J. Clin. Nutr..

[21] Hubbard T.D., Murray I.A., Bisson W.H., Lahoti T.S., Gowda K., Amin S.G., Patterson A.D., Perdew G.H. (2015). Adaptation of the human aryl hydrocarbon receptor to sense microbiota-derived indoles. Sci. Rep..

[22] Sartor R.B. (2006). Mechanisms of disease: Pathogenesis of Crohn’s disease and ulcerative colitis. Nat. Clin. Pract. Gastroenterol. Hepatol..

[23] Zelante T., Iannitti R.G., Cunha C., De Luca A., Giovannini G., Pieraccini G., Zecchi R., D’Angelo C., Massi-Benedetti C., Fallarino F. (2013). Tryptophan catabolites from microbiota engage aryl hydrocarbon receptor and balance mucosal reactivity via interleukin-22. Immunity.

[24] Tomii A., Takei C., Yoshikiyo K., Shimizu H. (2025). The Gut Microbial Metabolite Indole-3-Acetic Acid Reprograms Systemic Homeostasis and Ameliorates IBD-Associated Cachexia Independent of Food Intake. Int. J. Mol. Sci..

[25] Chowdhury M.M.I., Tomii A., Ishii K., Tahara M., Hitsuda Y., Koto Y., Kurata K., Yuasa K., Nishimura K., Shimizu H. (2021). TLR4 may be a novel indole-3-acetic acid receptor that is implicated in the regulation of CYP1A1 and TNFα expression depending on the culture stage of Caco-2 cells. Biosci. Biotechnol. Biochem..

[26] Tomii A., Higa M., Naito K., Kurata K., Kobayashi J., Takei C., Yuasa K., Koto Y., Shimizu H. (2023). Activation of the TLR4-JNK but not the TLR4-ERK pathway induced by indole-3-acetic acid exerts anti-proliferative effects on Caco-2 cells. Biosci. Biotechnol. Biochem..

[27] Liu D., Wei Y., Liu X., Zhou Y., Jiang L., Yin J., Wang F., Hu Y., Nanjaraj Urs A.N., Liu Y. (2018). Indoleacetate decarboxylase is a glycyl radical enzyme catalysing the formation of malodorant skatole. Nat. Commun..

[28] Whitehead T.R., Price N.P., Drake H.L., Cotta M.A. (2008). Catabolic pathway for the production of skatole and indoleacetic acid by the acetogen Clostridium drakei, Clostridium scatologenes, and swine manure. Appl. Environ. Microbiol..

[29] Claus R., Raab S. (1999). Influences on skatole formation from tryptophan in the pig colon. Adv. Exp. Med. Biol..

[30] Zgarbová E., Vrzal R. (2023). Skatole: A thin red line between its benefits and toxicity. Biochimie.

[31] Ruangyuttikarn W., Appleton M.L., Yost G.S. (1991). Metabolism of 3-methylindole in human tissues. Drug Metab. Dispos..

[32] Lanza D.L., Yost G.S. (2001). Selective dehydrogenation/oxygenation of 3-methylindole by cytochrome P450 enzymes. Drug Metab. Dispos..

[33] Kurata K., Kawahara H., Nishimura K., Jisaka M., Yokota K., Shimizu H. (2019). Skatole regulates intestinal epithelial cellular functions through activating aryl hydrocarbon receptors and p38. Biochem. Biophys. Res. Commun..

[34] Ishii K., Naito K., Tanaka D., Koto Y., Kurata K., Shimizu H. (2024). Molecular Mechanisms of Skatole-Induced Inflammatory Responses in Intestinal Epithelial Caco-2 Cells: Implications for Colorectal Cancer and Inflammatory Bowel Disease. Cells.

[35] Kurata K., Ishii K., Koto Y., Naito K., Yuasa K., Shimizu H. (2023). Skatole-induced p38 and JNK activation coordinately upregulates, whereas AhR activation partially attenuates TNFα expression in intestinal epithelial cells. Biosci. Biotechnol. Biochem..

[36] Safe S., Jin U.H., Park H., Chapkin R.S., Jayaraman A. (2020). Aryl Hydrocarbon Receptor (AHR) Ligands as Selective AHR Modulators (SAhRMs). Int. J. Mol. Sci..

[37] Schopf F.H., Biebl M.M., Buchner J. (2017). The Hsp90 chaperone machinery. Nat. Rev. Mol. Cell Biol..

[38] Katayama K., Noguchi K., Sugimoto Y. (2013). FBXO15 regulates P-glycoprotein/ABCB1 expression through the ubiquitin–proteasome pathway in cancer cells. Cancer Sci..

[39] Luecke-Johansson S., Gralla M., Rundqvist H., Ho J.C., Johnson R.S., Gradin K., Poellinger L. (2017). A Molecular Mechanism To Switch the Aryl Hydrocarbon Receptor from a Transcription Factor to an E3 Ubiquitin Ligase. Mol. Cell. Biol..

[40] Ichisaka Y., Yano S., Nishimura K., Niwa T., Shimizu H. (2024). Indoxyl sulfate contributes to colorectal cancer cell proliferation and increased EGFR expression by activating AhR and Akt. Biomed. Res..

[41] Ichisaka Y., Takei C., Naito K., Higa M., Yano S., Niwa T., Shimizu H. (2025). The Role of Indoxyl Sulfate in Exacerbating Colorectal Cancer During Chronic Kidney Disease Progression: Insights into the Akt/β-Catenin/c-Myc and AhR/c-Myc Pathways in HCT-116 Colorectal Cancer Cells. Toxins.

[42] Ohgane K., Yoshioka H. (2019). Quantification of gel bands by an image J macro, band/peak quantification tool. Protocols.io.

[43] Denison M.S., Soshilov A.A., He G., DeGroot D.E., Zhao B. (2011). Exactly the same but different: Promiscuity and diversity in the molecular mechanisms of action of the aryl hydrocarbon (dioxin) receptor. Toxicol. Sci..

[44] Rasmussen M.K., Balaguer P., Ekstrand B., Daujat-Chavanieu M., Gerbal-Chaloin S. (2016). Skatole (3-Methylindole) Is a Partial Aryl Hydrocarbon Receptor Agonist and Induces CYP1A1/2 and CYP1B1 Expression in Primary Human Hepatocytes. PLoS ONE.

[45] Jin U.H., Lee S.O., Sridharan G., Lee K., Davidson L.A., Jayaraman A., Chapkin R.S., Alaniz R., Safe S. (2014). Microbiome-derived tryptophan metabolites and their aryl hydrocarbon receptor-dependent agonist and antagonist activities. Mol. Pharmacol..

[46] Vyhlídalová B., Krasulová K., Pečinková P., Marcalíková A., Vrzal R., Zemánková L., Vančo J., Trávníček Z., Vondráček J., Karasová M. (2020). Gut Microbial Catabolites of Tryptophan Are Ligands and Agonists of the Aryl Hydrocarbon Receptor: A Detailed Characterization. Int. J. Mol. Sci..

[47] Li M., Ding Y., Wei J., Dong Y., Wang J., Dai X., Yan J., Chu F., Zhang K., Meng F. (2024). Gut microbiota metabolite indole-3-acetic acid maintains intestinal epithelial homeostasis through mucin sulfation. Gut Microbes.

[48] Soshilov A., Denison M.S. (2011). Ligand displaces heat shock protein 90 from overlapping binding sites within the aryl hydrocarbon receptor ligand-binding domain. J. Biol. Chem..

[49] Akkaya B.G., Zolnerciks J.K., Ritchie T.K., Bauer B., Hartz A.M., Sullivan J.A., Linton K.J. (2015). The multidrug resistance pump ABCB1 is a substrate for the ubiquitin ligase NEDD4-1. Mol. Membr. Biol..

[50] Folkes L.K., Wardman P. (2001). Oxidative activation of indole-3-acetic acids to cytotoxic species-A potential new role for plant auxins in cancer therapy. Biochem. Pharmacol..

[51] Amoozadeh Y., Dan Q., Xiao J., Waheed F., Szászi K. (2015). Tumor necrosis factor-alpha induces a biphasic change in claudin-2 expression in tubular epithelial cells: Role in barrier functions. Am. J. Physiol. Cell Physiol..

[52] Shi J., Zhao D., Song S., Zhang M., Zamaratskaia G., Xu X., Zhou G., Li C. (2020). High-Meat-Protein High-Fat Diet Induced Dysbiosis of Gut Microbiota and Tryptophan Metabolism in Wistar Rats. J. Agric. Food Chem..

[53] Babich V., Vadnagara K., Di Sole F. (2013). The biophysical and molecular basis of intracellular pH sensing by Na+/H+ exchanger-3. FASEB J..

[54] He P., Yun C.C. (2010). Mechanisms of the regulation of the intestinal Na+/H+ exchanger NHE3. J. Biomed. Biotechnol..

[55] Le Leu R.K., Brown I.L., Hu Y., Morita T., Esterman A., Young G.P. (2007). Effect of dietary resistant starch and protein on colonic fermentation and intestinal tumourigenesis in rats. Carcinogenesis.

[56] Hawe S.M., Walker N., Moss B.W. (1992). The effects of dietary fibre, lactose and antibiotic on the levels of skatole and indole in faeces and subcutaneous fat in growing pigs. Anim. Sci..

[57] Krishnan S., Ding Y., Saedi N., Choi M., Sridharan G.V., Sherr D.H., Yarmush M.L., Alaniz R.C., Jayaraman A., Lee K. (2018). Gut Microbiota-Derived Tryptophan Metabolites Modulate Inflammatory Response in Hepatocytes and Macrophages. Cell Rep..

