# Supplementary material for: Indole-3-Acetic Acid and Skatole Exert Opposing Effects on MDR1 Proteostasis in Human Colonic Epithelial Cells: A Molecular Basis for the Gut Microbial Metabolic Switch
Source: J Xenobiot. 2026 Feb 18;16(1):36. doi: 10.3390/jox16010036 (PMC12942598; doi:10.3390/jox16010036)
Supplement: Supplementary file 1 [file jox-16-00036-s001.zip › jox-4105613-supplementary.pdf]

Journal of *Xenobiotics*

MDPI

Supplementary Materials: Indole-3-acetic acid and Skatole Exert Opposing Effects on MDR1 Proteostasis via Proposed AhR Structural Antagonism: Implications for the Gut Microbial Metabolic Switch

Kazuma Naito, Ayame Tomii, Katsunori Ishii, Hidehisa Shimizu

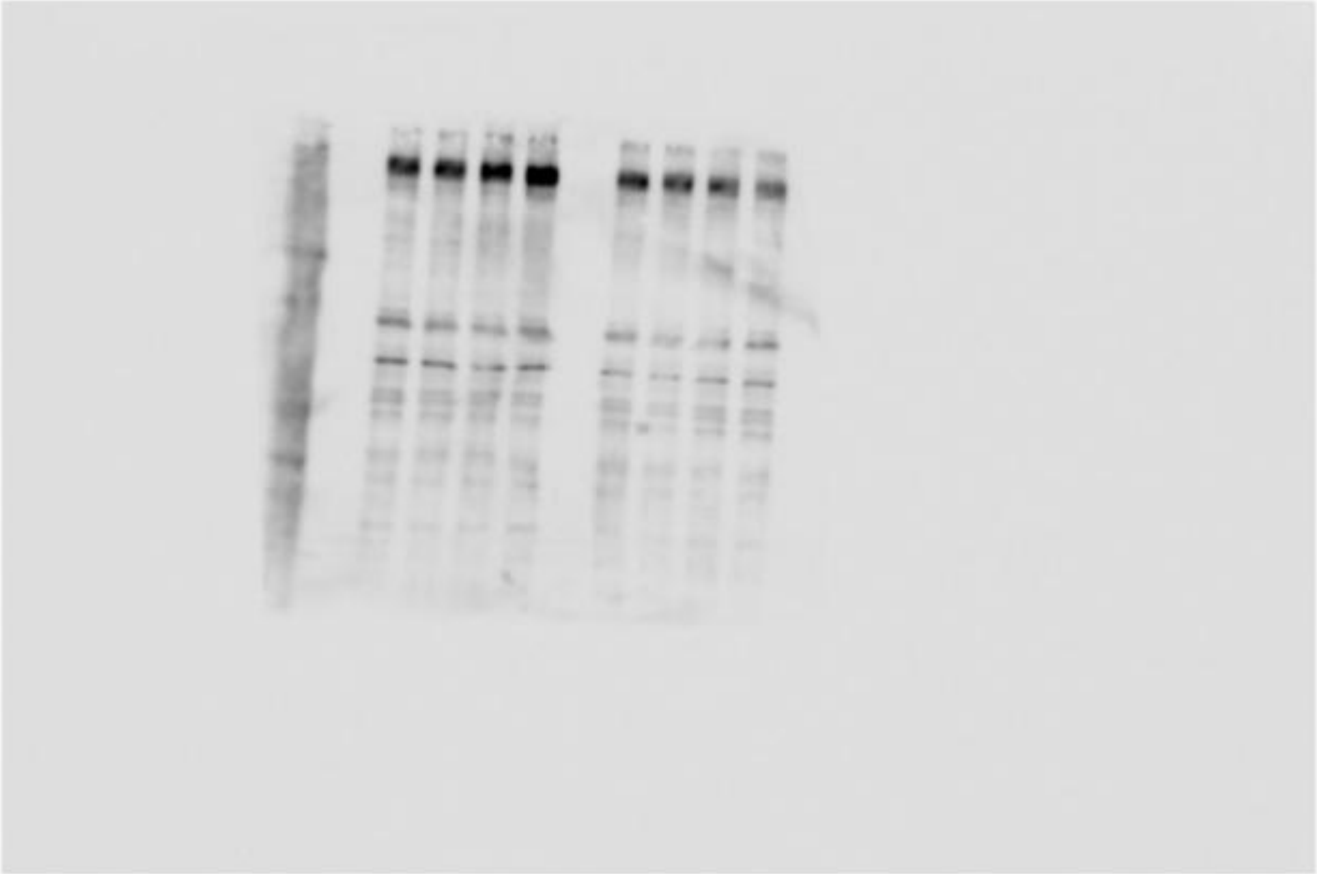

Figure 1A and B. Naito et al.
(MDR1)

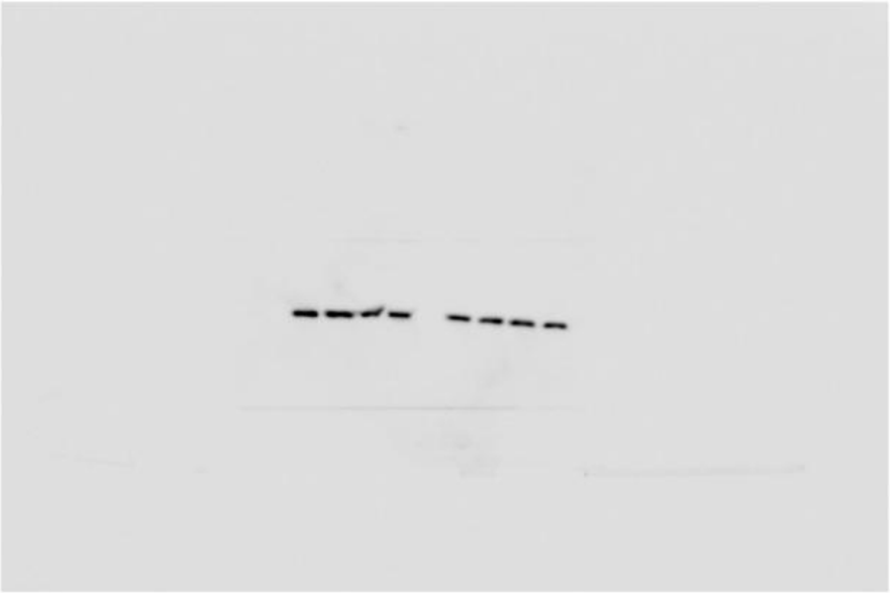

Figure 1A and B. Naito et al.
(β-actin)

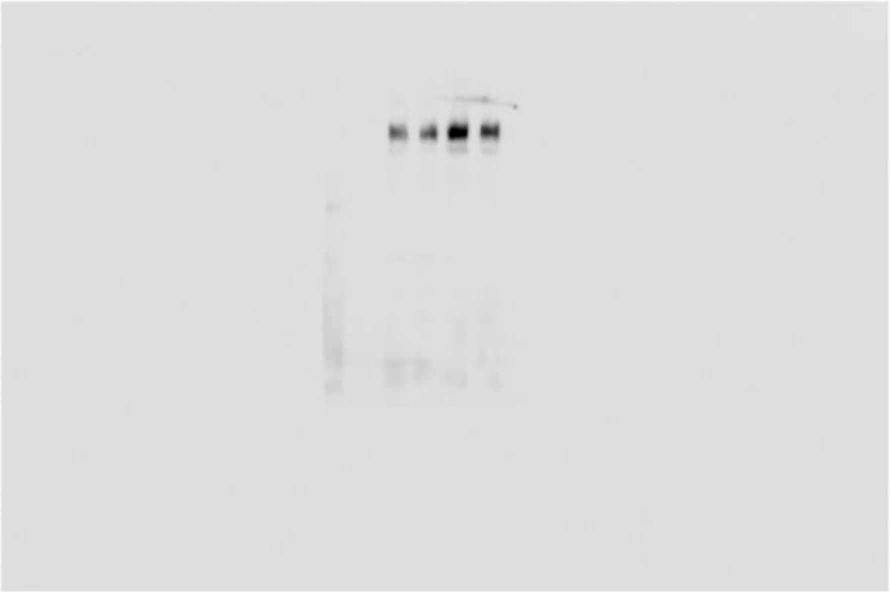

Figure 3. Naito et al.
(MDR1)

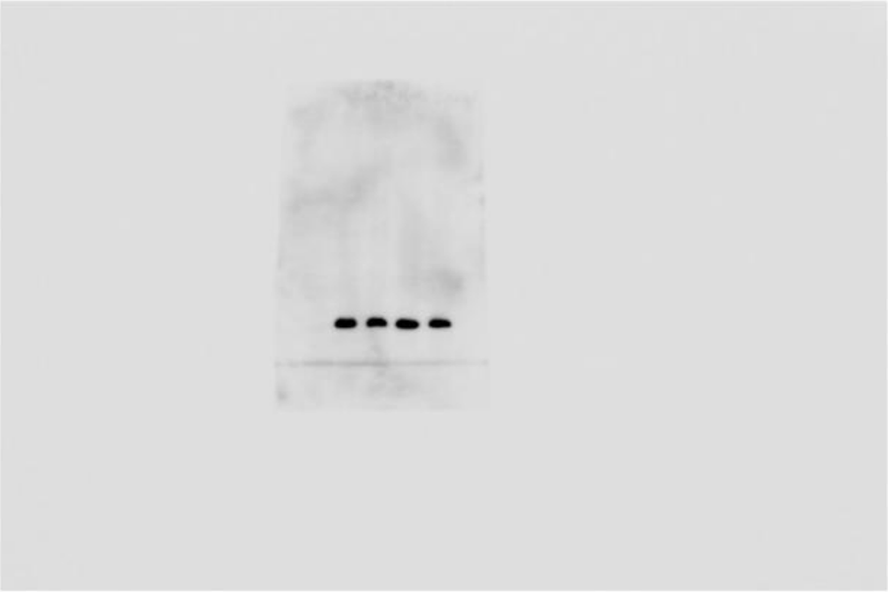

Figure 3. Naito et al.
(β-actin)

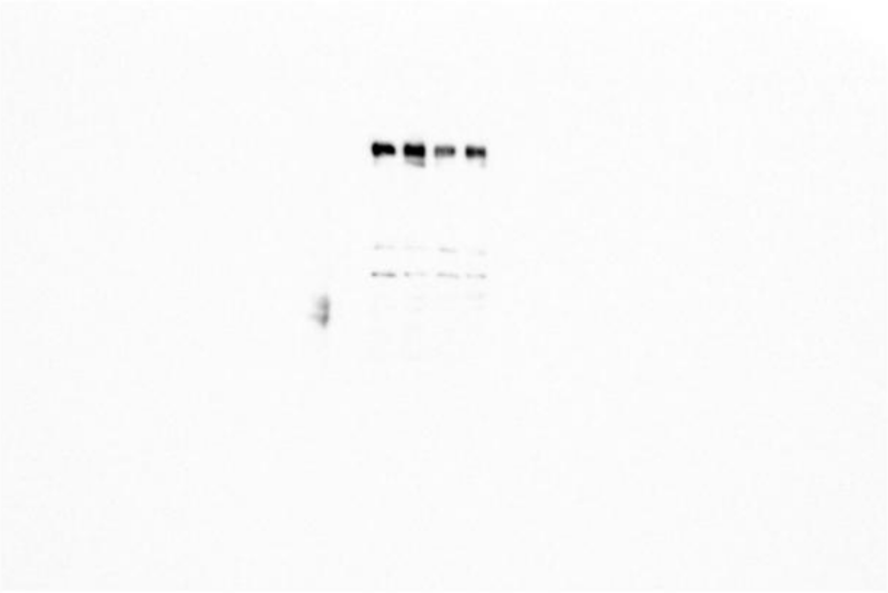

Figure 4. Naito et al.
(MDR1)

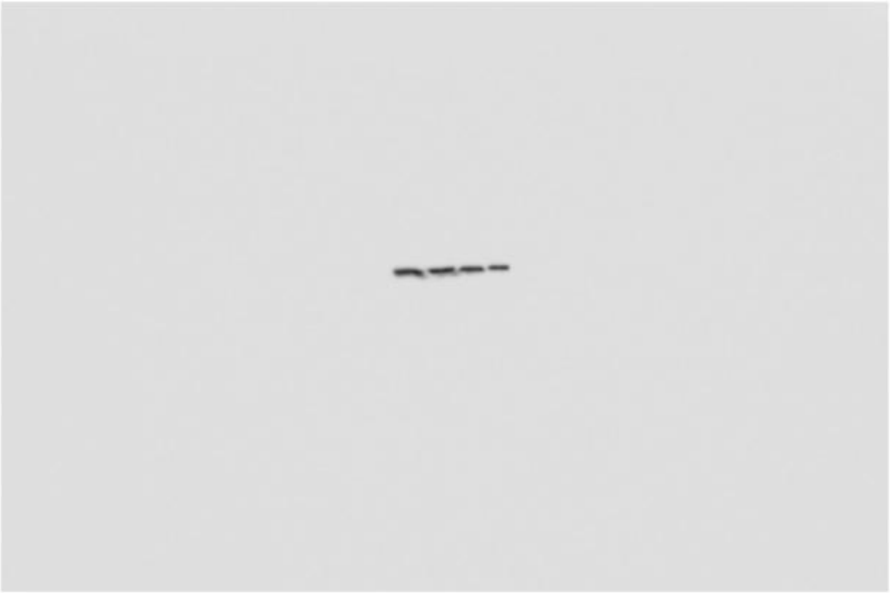

Figure 4. Naito et al.
(β-actin)
